# Behavioral Changes in Mice Lacking Interleukin-33

**DOI:** 10.1523/ENEURO.0147-17.2017

**Published:** 2017-12-26

**Authors:** Eisuke Dohi, Eric Y. Choi, Indigo V.L. Rose, Akiho S. Murata, Sharon Chow, Minae Niwa, Shin-ichi Kano

**Affiliations:** Department of Psychiatry and Behavioral Sciences, The Johns Hopkins University School of Medicine, Baltimore, MD 21287

**Keywords:** anxiety, astrocytes, cytokines, IL-33, oligodendrocytes, social behavior

## Abstract

Interleukin (IL)-33 is a member of the IL-1 family of cytokines. IL-33 is expressed in nuclei and secreted as alarmin upon cellular damage to deliver a danger signal to the surrounding cells. Previous studies showed that IL-33 is expressed in the brain and that it is involved in neuroinflammatory and neurodegenerative processes in both humans and rodents. Nevertheless, the role of IL-33 in physiological brain function and behavior remains unclear. Here, we have investigated the behaviors of mice lacking IL-33 (*Il33* ^−/−^ mice). IL-33 is constitutively expressed throughout the adult mouse brain, mainly in oligodendrocyte-lineage cells and astrocytes. Notably, *Il33* ^−/−^ mice exhibited reduced anxiety-like behaviors in the elevated plus maze (EPM) and the open field test (OFT), as well as deficits in social novelty recognition, despite their intact sociability, in the three-chamber social interaction test. The immunoreactivity of c-Fos proteins, an indicator of neuronal activity, was altered in several brain regions implicated in anxiety-related behaviors, such as the medial prefrontal cortex (mPFC), amygdala, and piriform cortex (PCX), in *Il33* ^−/−^ mice after the EPM. Altered c-Fos immunoreactivity in *Il33* ^−/−^ mice was not correlated with IL-33 expression in wild-type (WT) mice nor was IL-33 expression affected by the EPM in WT mice. Thus, our study has revealed that *Il33* ^−/−^ mice exhibit multiple behavioral deficits, such as reduced anxiety and impaired social recognition. Our findings also indicate that IL-33 may regulate the development and/or maturation of neuronal circuits, rather than control neuronal activities in adult brains.

## Visual Abstract

**Figure:**
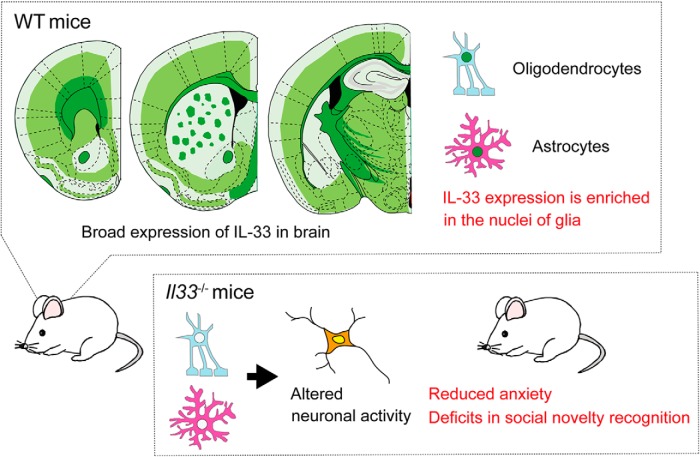

## Significance Statement

Interleukin (IL)-33 is expressed in the developing and mature brain; however, its role in brain function and behavior remains unclear. Here, we report that mice lacking IL-33 (*Il33* ^−/−^ mice) showed reduced anxiety-like behaviors and impaired social novelty recognition. In *Il33* ^−/−^ mice, c-Fos immunoreactivity was altered in several brain areas, including those related to anxiety, after a stressful behavioral assay. Notably, IL-33 expression was not correlated with c-Fos immunoreactivity, and no IL-33 expression changes were seen after the behavioral assay. Thus, our study suggests that *Il33* deficiency impairs multiple behaviors, such as anxiety and social behaviors, by altering brain development/maturation. These findings will advance our understanding of the physiological role of immune molecules in brain function and behavior.

## Introduction

Interleukin (IL)-33 is a member of the IL-1 family of cytokines, which contains 11 members, including IL-1α, IL-1β, and IL-18 (Molofsky et al., 2015; Liew et al., 2016). IL-33 is expressed in the nuclei of various types of cells and is also secreted as a cytokine, particularly by barrier cells such as endothelial and epithelial cells, as a signal of cellular injury and necrosis (Molofsky et al., 2015; Liew et al., 2016; Martin and Martin, 2016). Thus, IL-33 is categorized as a nuclear alarmin, an endogenous molecule that is released on tissue injury to promote innate immune responses, similar to high-mobility group box 1 protein (HMGB1) and IL-1α. Upon secretion, IL-33 binds to the IL-33 receptor ST2 on its target cells and recruits IL-1 receptor accessory proteins (IL1RAcPs) to form a heterotrimeric signaling complex. This complex activates NF-κB, ERK 1/2, JNK, and p38 MAPK signaling to induce the expression of downstream genes (Molofsky et al., 2015; Liew et al., 2016; Martin and Martin, 2016). IL-33 can also function as a transcriptional modulator (Molofsky et al., 2015; Liew et al., 2016; Martin and Martin, 2016). IL-33 contains a nuclear localization sequence and a DNA-binding domain at the N-terminus and binds to the nucleosome acidic pocket formed by the histone H2A-H2B dimer (Roussel et al., 2008). IL-33 also binds to the histone methyltransferase SUV39H1 and NF-κB, modulating their activity (Ali et al., 2011). Nevertheless, the precise mechanisms by which IL-33 regulates gene expression in various cell types remain to be elucidated.

IL-33 is expressed in the central nervous system (Schmitz et al., 2005; Wicher et al., 2013; Gadani et al., 2015). The original report on the discovery of IL-33 showed that mRNA expression was most abundant in the brain and spinal cord (Schmitz et al., 2005). Subsequent studies added that the pattern of IL-33 expression changes in a temporal and spatial manner during brain development, with its expression enriched in astrocytes in the mature brain (Wicher et al., 2013). Furthermore, a recent report revealed that IL-33 is predominantly expressed in postmitotic oligodendrocytes in the white matter (Gadani et al., 2015). Roles of IL-33 in neuroinflammation and neurodegeneration have been reported as well (Chapuis et al., 2009; Jiang et al., 2012; Yu et al., 2012; Xiong et al., 2014; Gadani et al., 2015; Fu et al., 2016). Human genetic studies reported that a single-nucleotide polymorphism (SNP) in IL-33 is associated with increased risk for the late-onset form of Alzheimer’s disease (Chapuis et al., 2009; Yu et al., 2012). Expression levels of IL-33 and its receptor ST2 are strongly increased around the amyloid plaques in the brains of patients with Alzheimer’s disease (Xiong et al., 2014). Furthermore, rodent studies showed that IL-33 had a therapeutic effect in a mouse model of Alzheimer disease, attenuating cellular pathologies and recovering behavioral phenotypes (Fu et al., 2016). Anti-inflammatory and potential therapeutic roles of IL-33 were also suggested by findings from mouse models of spinal cord injury and experimental autoimmune encephalitis (Jiang et al., 2012; Gadani et al., 2015). Taken together, these findings highlight the significant role of glial IL-33 in neurodegeneration, injury, and inflammation in the brain. Nevertheless, the physiological role of IL-33 in brain function and behavior remains poorly understood.

In this study, we provide evidence that IL-33 is involved in anxiety-related and social behaviors. In the elevated plus maze (EPM) and the open field test (OFT), mice lacking IL-33 (*Il33* ^−/−^ mice) exhibited reduced anxiety-like behaviors compared to wild-type (WT) mice. In the three-chamber social interaction test, *Il33* ^−/−^ mice showed deficits in social novelty recognition, despite their intact sociability. In the adult mouse brain, IL-33 expression was detected in the cellular nuclei of various brain regions including the cortex, corpus callosum (CC), hypothalamus, amygdala, and hippocampus. The expression of IL-33 in these brain regions was localized primarily to Olig2-expressing oligodendrocyte-lineage cells and, to a lesser extent, S100β-expressing astrocytes. Assessment of neuronal activity by analyzing c-Fos expression in neurons immediately after the EPM suggested altered neuronal activities in multiple brain regions, including those related to anxiety in *Il33* ^−/−^ mice. Nonetheless, altered c-Fos immunoreactivity in *Il33* ^−/−^ mice was not correlated with IL-33 expression in WT mice nor was IL-33 expression affected by the EPM in WT mice. Thus, our study suggested that *Il33* deficiency results in multiple behavioral deficits, such as reduced anxiety and impaired social novelty recognition, possibly via dysregulated developmental and/or maturation of multiple neuronal circuits.

## Materials and Methods

### Animals

*Il33* ^−/−^ mice on a C57BL/6J background were provided by Amgen Inc. C57BL/6J mice were purchased from The Jackson Laboratory. Mice were housed in a specific pathogen-free animal facility under controlled temperatures (23 ± 3°C) and controlled light schedule (lights on from 7 A.M. to 9 P.M.), with food and water available. Unless stated otherwise, male mice were used for the experiments at 8–10 weeks of age. All experimental procedures were performed in accordance with the National Institutes of Health Guidelines for the Care and Use of Laboratory Animals, under the animal protocols approved by the Institutional Animal Care and Use Committee.

### Behavioral assays

Behavioral assays were performed on male mice at 8–10 weeks of age, following the established protocols. All the assays were conducted between 10 A.M. and 3 P.M. during the light phase. Two independent cohorts of mice were used in the present study. In the first cohort, mice were tested in the OFT and then in the elevated-plus maze on the following day. In the second cohort, mice were assessed in the three-chamber social interaction test.

#### OFT

Novelty-induced activity in the open field was assessed as described previously (Niwa et al., 2010; Zhang et al., 2011; Dubal et al., 2015; Ertürk et al., 2016; Xiong et al., 2017). Locomotion, rearing, and center time were measured for 10 min using a Photobeam Activity System (PAS–Open Field, San Diego Instruments). The PAS system consisted of two vertically stacked frames, each containing infrared lasers arranged in a 16 × 16 grid, which detected mouse movement, including ambulation and rearing. The open field box and surrounding photobeam apparatus were housed in a ventilated cabinet. Single-beam breaks were automatically recorded as “counts” and the PAS system automatically started recording counts once the mouse started moving. The total counts were recorded and the percentages of center counts (defined as those in the central 27.5 × 27.5 cm area) to total counts were calculated.

#### EPM

General anxiety was evaluated by the EPM as described previously (Walf and Frye, 2007; Sidor et al., 2010; Montalvo-Ortiz et al., 2016). The maze consisted of two closed arms, with walls of height 20 cm, and two open arms extending from a central platform, raised to a height of 40 cm above the floor. Mice were placed in the central platform of the maze facing an open arm and allowed to explore the maze for 5 min. The EPM was conducted in the light condition (at 150 lux) so mice could perceive the difference between open and closed arms. Mouse activity was recorded on videotape and the following measurements were manually quantified: total number of entries into the arms, number of entries into closed/open arms (defined as all four limbs entering the arm), and total time spent in closed/open arms.

#### Three-chamber social interaction test

The three-chamber social interaction test was conducted as previously described (Moy et al., 2004; Gkogkas et al., 2013; Lipina et al., 2013; Filiano et al., 2016). All mice were tested in a nonautomated three-chambered box. Dividing walls had retractable doorways allowing access into each chamber. Mice were acclimated to the three-chambered box for 4 d before the test (10 min/d). On the test day, mice were transported to the testing room and habituated for at least 1 h before the experiment. A white noise generator was used to mitigate any unforeseen noises. The subject mouse was habituated in the chamber with two empty cylinders for 10 min. Then, the “toy” object was placed in one of the cylinders and mouse (stranger 1) was placed in another cylinder for the “sociability” trial. Mice were allowed to explore the chambers for 10 min. In the next “social novelty preference” trial, stranger 1 was kept in the cylinder as the familiar mouse and the toy object was replaced with a novel mouse (stranger 2). The subject mouse was again allowed to explore the chambers. During these two trials, mouse activities were recorded on video and the time spent sniffing each cylinder was manually measured. Preference index was calculated as follows. For sociability test: (sniffing time to mouse) × 100/(sniffing time to mouse + sniffing time to object) − 50. For social novelty preference test: (sniffing time to novel mouse) × 100/(sniffing time to novel mouse + sniffing time to familiar mouse) − 50.

#### Assessment of inter-rater reliability

In the EPM and three-chamber social interaction test, two examiners blinded to genotype followed a rigorous protocol with which highly reproducible results were obtained previously in our group. Inter-rater reliability was assessed by intraclass correlation coefficient (ICC; Shrout and Fleiss, 1979; Langford et al., 2010; Hallgren, 2012; Koo and Li, 2016). ICC estimates and their 95% confident interval were calculated using SPSS statistical package version 25 (SPSS Inc) with the following parameters: mean-rating (k = 2), absolute-agreement, two-way random effects model. All data displayed high inter-rater reliability, with ICC rating ranged from 0.939 to 0.984 (Table 1).

**Table 1.** ICC

Figure	Data structure	Type of test	Sample size	Statistical data
Fig. 2A–C	Normal distribution	Intraclass correlation coefficient	*n* = 15 (elevated-plus maze test)	Time spent in open arm; ICC = 0.98 (95%CI 0.94–0.993)Open arm entries; ICC = 0.97 (95%CI 0.898–0.99)Total entries; ICC = 0.94 (95%CI 0.818–0.98)
Fig. 5A,C	Normal distribution	Intraclass correlation coefficient	Sniffing time (three-chamber ocial interaction test); *n* = 64 (from 16 mice)	Sniffing time; ICC = 0.983 (95%CI 0.971–0.989)
Fig. 5B,D	Normal distribution	Intraclass correlation coefficient	Preference index (three-chamber social interaction test); *n* = 16	Preference index (sociability); ICC = 0.987 (95%CI 0.939–0.992)Preference index (social novelty preference); ICC = 0.939 (95%CI 0.806–0.98)

**Table 2.** Statistical table

Figure	Data structure	Type of test	Sample size	Statistical data
Fig. 1F	Normal distribution	Unpaired **t**test (two-tailed)	Olig2^+^: *n* = 5S100β^+^: *n* = 5	*t* = 3.932, df = 8, *p* = 0.0043Cohen’s *d* = 2.487
Fig. 1H	Normal distribution	One-way ANOVA	WT mice: *n* = 2 (three to five images/mouse)	F = 3.328, *p* = 0.0134, η^2^ = 0.299Tukey’s multiple comparison test:mPFC vs M2: *p* = 0.8229, Cohen’s *d* = 0.523mPFC vs CC: *p* = 0.0441, Cohen’s *d* = 1.79mPFC vs Pe: *p* > 0.9999, Cohen’s *d* = 0.115mPFC vs BLA: *p* = 0.9835, Cohen’s *d* = 0.355mPFC vs CoA: *p* = 0.9934, Cohen’s *d* = 0.236M2 vs CC: *p* = 0.4619, Cohen’s *d* =1.027M2 vs Pe: *p* = 0.9441, Cohen’s *d* = 0.435M2 vs BLA: *p* = 0.4175, Cohen’s *d* = 0.962M2 vs CoA: *p* = 0.9882, Cohen’s *d* = 0.262CC vs Pe: *p* = 0.1238, Cohen’s *d* = 1.791CC vs BLA: *p* = 0.0076, Cohen’s *d* = 3.228CC vs CoA: *p* = 0.1843, Cohen’s *d* = 1.309Pe vs BLA: *p* = 0.954, Cohen’s *d* = 0.52Pe vs CoA: *p* = 0.9997, Cohen’s *d* = 0.602BLA vs CoA: *p* = 0.8305, Cohen’s *d* = 0.139
Fig. 1I*	Normal distribution	Kruskal-Wallis test	WT mice: *n* = 2 (three to five images/mouse)	*H* = 19.22, *p* = 0.0007, ε2 = 0.534Dunnett’s multiple comparison test:mPFC vs M2: *p* > 0.9999, Cohen’s *d* = 0.353mPFC vs Pe: *p* = 0.4307, Cohen’s *d* = 1.229mPFC vs BLA: *p* = 0.0462, Cohen’s *d* = 1.81mPFC vs CoA: *p* = 0.1536, Cohen’s *d* = 1.354M2 vs Pe: *p* = 0.0929, Cohen’s *d* = 2.09M2 vs BLA: *p* = 0.0055, Cohen’s *d* = 3.246M2 vs CoA: *p* = 0.0247, Cohen’s *d* = 2.026Pe vs BLA: *p* > 0.9999, Cohen’s *d* = 0.489Pe vs CoA: *p* > 0.9999, Cohen’s *d* = 0.272BLA vs CoA: *p* > 0.9999, Cohen’s *d* = 0.127
Fig. 2A	Normal distribution	Unpaired **t**test (two-tailed)	WT mice: *n* = 12*Il33*^−/−^ mice *n* = 13	*t* = 6.911, df = 23, *p* < 0.0001Cohen’s *d* = 2.794
Fig. 2B	Normal distribution	Unpaired **t**test (two-tailed)	WT mice: *n* = 12*Il33*^−/−^ mice *n* = 13	*t* = 6.923, df = 23, *p* < 0.0001Cohen’s *d* = 2.701
Fig. 2C	Normal distribution	Unpaired **t**test (two-tailed)	WT mice: *n* = 12*Il33*^−/−^ mice *n* = 13	*t* = 0.1446, df = 23, *p* = 0.8863Cohen’s *d* = 0.058
Fig. 2D	Normal distribution	Unpaired **t**test (two-tailed)	WT mice: *n* = 9*Il33*^−/−^ mice *n* = 7	*t* = 4.447, df = 14, *p* = 0.0006Cohen’s *d* = 2.280
Fig. 3B	Normal distribution	Unpaired **t**test (two-tailed)	WT mice: *n* = 4*Il33*^−/−^ mice *n* = 3–4	mPFC; *t* = 2.972, df = 6, *p* = 0.0249, Cohen’s *d* = 2.103PCX; *t* = 3.401, df = 6, *p* = 0.0145, Cohen’s *d* = 2.406M2; *t* = 2.462, df = 6, *p* = 0.0490, Cohen’s *d* = 1.741S1BF; *t* = 8.439, df = 6, *p* = 0.0002, Cohen’s *d* = 5.697BLA; *t* = 1.885, df = 6, *p* = 0.2047, Cohen’s *d* = 1.334CeA; *t* = 3.149, df = 5, *p* = 0.0254, Cohen’s *d* = 2.177vHip; *t* = 1.414, df = 5, *p* = 0.2107, Cohen’s *d* = 3.071
Fig. 4A	Normal distribution	Pearson’s *r*	WT mice: *n* = 5*Il33*^−/−^ mice *n* = 5IL-33 in WT mouse fold change of c-Fos in WT mice normalized with *Il33* ^−/−^ mice	*r* = 0.1295 (95% CI −0.28 to 0.4991)R^2^ = 0.01677*p* = 0.5373
Fig. 4B	Normal distribution	Pearson’s *r*	WT mice: *n* = 5	*r* = −0.07765 (95% CI −0.4587 to 0.3275)R^2^ = 0.006029*p* = 0.7122
Fig. 4C	Normal distribution	Unpaired **t**test (two-tailed)	Ctr: *n* = 8 imagesPost-EPM: *n* = 8 images (two images/mouse)	*t* = 0.03388, df = 14, *p* = 0.9735Cohen’s *d* = 0.017
Fig. 4D	Normal distribution	Unpaired **t**test (two-tailed)	Ctr: *n* = 3 imagesPost-EPM: *n* = 4 images (one images/mouse)	*t* = 0.2921, df = 5, *p* = 0.7820Cohen’s *d* = 0.221
Fig. 4E	Normal distribution	Unpaired **t**test (two-tailed)	Ctr: *n* = 4Post-EPM: *n* = 4	*t* = 0.3453, df = 6, *p* = 0.7416Cohen’s *d* = 0.224
Fig. 5A	Two-factors (genotype and chamber)	Two-way ANOVA with RM	WT mice: *n* = 15*Il33*^−/−^ mice: *n* = 9	Genotype: *F*_(1,22)_ = 0.09669, *p* = 0.7588, η^2^ = 0.009Mouse/Object: *F*_(1,22)_ = 54.44, *p* < 0,0001Interaction: *F*_(1,22)_ = 0.9178, *p* = 0.3485Sidak’s multiple comparisons test:Mouse/Object, WT: *p* < 0.0001Mouse/Object, *Il33*^−/−^: *p* < 0.0001
Fig. 5B	Normal distribution	Unpaired **t**test (two-tailed)	WT mice: *n* = 15*Il33*^−/−^ mice: *n* = 9	*t* = 0.9374, df = 22, *p* = 0.3587Cohen’s *d* = 0.395
Fig. 5C	Two-factors (genotype and chamber)	Two-way ANOVA with RM	WT mice: *n* = 15*Il33*^−/−^ mice: *n* = 9	Genotype: *F*_(1,22)_ = 0.0224, *p* = 0.8824, η^2^ = 0.043Familiar/Novel: *F*_(1,22)_ =26.34, *p* < 0,0001Interaction: *F*_(1,22)_ = 4.681, *p* = 0.0416Sidak’s multiple comparisons test:Familiar/Novel, WT: *p* < 0.0001Familiar/Novel, *Il33*^−/−^: *p* = 0.1420
Fig. 5D	Normal distribution	Unpaired **t**test (two-tailed)	WT mice: *n* = 15*Il33*^−/−^ mice: *n* = 9	*t* = 2.407, df = 22, *p* = 0.0249Cohen’s *d* = 1.085

**Figure 1.**
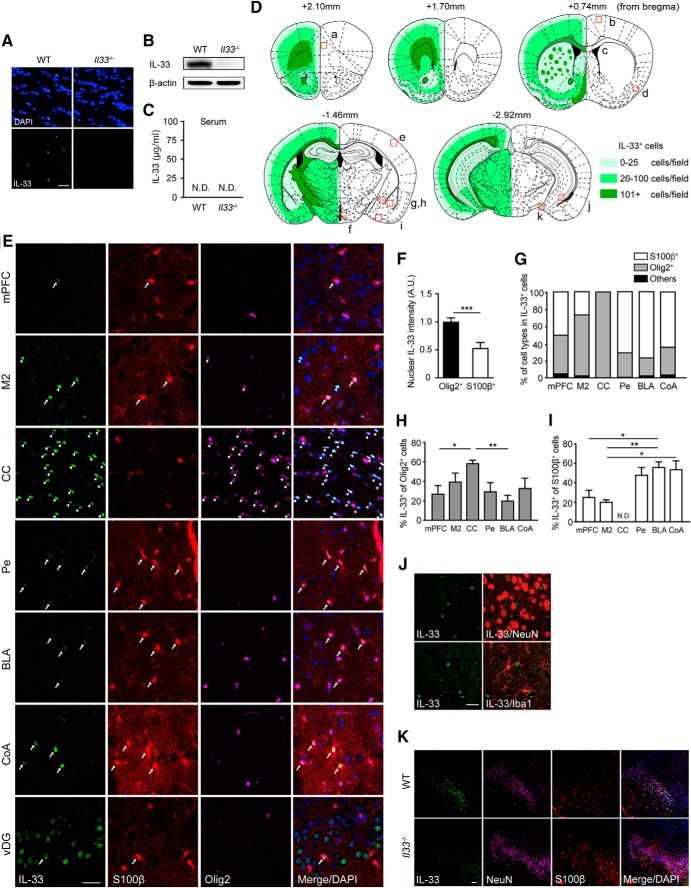
IL-33 expression patterns in the adult mouse brain. ***A***, Validation of specificity of IL-33 immunostaining using brain sections (CC) from WT and *Il33* ^−/−^ mice. ***B***, Western blot analysis of IL-33 expression in the cortex from WT and *Il33* ^−/−^ mice. β-actin was used as an internal control for protein loading. ***C***, Absence of IL-33 proteins in the peripheral blood of WT mice. The levels of serum IL-33 proteins were below the detection threshold of ELISA assay (15.6 pg/ml). N.D., not detected. ***D***, Schematic illustration of the distribution of IL-33-expressing cells. The number of IL-33-expressing cells was quantified at 20× under a fluorescence microscope. Light, medium, and dark green correspond to 0–25, 26–100, and 101+ cells per field, respectively. Small red boxes indicate the area analyzed in ***E–K*** and Figs. 3, 4*A–D* : ***a***, mPFC; ***b***, secondary motor cortex (M2); ***c***, CC; ***d***, PCX; ***e***, primary S1BF; ***f***, Pe; ***g***, CeA; ***h***, BLA; ***i***, CoA; ***j***, ventral hippocampus (vHip); ***k***, vDG. ***E***, Representative pictures of IL-33 expression in several brain regions. Arrows, IL-33^+^ astrocytes (S100β^+^ cells); arrowheads, IL-33^+^ oligodendrocyte-lineage cells (Olig2^+^ cells). ***F***, The average nuclear intensity of IL-33 between Olig2^+^ cells and S100β^+^ cells in the M2. A.U., arbitrary unit. ***G***, Percentages of S100β^+^, Olig2^+^, and other cells among IL-33^+^ cells in each brain region. ***H***, Comparison of IL-33 expression in Olig2^+^ cells across brain regions. ***I***, Comparison of IL-33 expression in S100β^+^ cells across brain regions. We also observed Olig2^+^ and S100β^+^ cells, but these cells are not included in this graph. ***J***, No colocalization of IL-33 signals to neurons (NeuN^+^ cells) or microglia (Iba1^+^ cells) in most brain regions. Representative pictures of the M2 are shown. ***K***, IL-33 colocalization to neurons in the granular layer of vDG. Scale bar, 30 µm. Each bar represents mean ± SEM; *p < 0.05, **p < 0.01 (Student’s *t* test, one-way ANOVA with *post hoc* Tukey’s test, and Kruskal–Wallis test with *post hoc* Dunnett’s test; see Table 2 for the detail of statistical analysis).

* In Fig. 1I, we did not include CC data for analysis because we could not detect IL-33^+^ S100β^+^ astrocytes in CC. In the Kruskal-Wallis test, we calculated ε^2^ for effect size: ε^2^ = *H*/((*n*^2^ − 1)/(*n* + 1)). *H*, Kruskal–Wallis test statistic value; *n*: total number of observations (King and Minium, 2003).

### Immunohistochemistry

Mice were anesthetized and transcardially perfused, first with ice-cold PBS and then with 4% paraformaldehyde (PFA) in PBS. The brains were removed and postfixed in the same fixative for 24 h, then incubated in 10% and 30% (w/v) sucrose in PBS. Free-floating coronal sections (40-µm thick) were prepared with a Cryostat (CM 3050S, Leica). For immunohistochemistry, the following primary antibodies were used: goat anti-mouse IL-33 (R&D Systems, RRID: AB_884269) at 1:200 dilution, mouse anti-NeuN (Merck Millipore, RRID: AB_2298772) at 1:500 dilution, rabbit anti-Iba1 (Wako Chemicals, RRID: AB_839504) at 1:400 dilution, rabbit anti-S100β (Abcam, RRID: AB_882426) at 1:400 dilution, goat anti-c-Fos (Santa Cruz Biotechnology, RRID: AB_2106783) at 1:500 dilution, and rabbit anti-c-Fos (Cell Signaling, RRID: AB_22472111) at 1:200 dilution. Fluorescently labeled secondary antibodies Alexa Fluor 488, 546, 633 at 1:400 dilution (Thermo Fisher Scientific) and DAPI (at 1:50,000 dilution, Roche) were used for signal detection. Images were acquired using Zeiss LSM510 and LSM700 confocal microscopes with Zen software (Carl Zeiss). Images were taken at 20× and 40× magnification. Areas used for quantification were indicated with red boxes. For c-Fos quantification, maximum projection images of serial z-stack sections (21 sections at 0.75 µm) were constructed using Zen software.

### Image analysis

Image analysis was performed as previously described (Gompf et al., 2010; Dohi et al., 2012; Gadani et al., 2015). Three to five brain sections from each animal were chosen based on anatomic landmarks to ensure that equivalent regions were analyzed. The number of IL-33-expressing cells per each visual field was quantified and compared across different brain regions. The color code of light, medium, and dark green correspond to 0–25, 26–100, and 101+ cells/visual field, respectively. To determine the difference in IL-33 expression level between Olig2^+^ and S100β^+^ cells, the IL-33^+^ nuclear intensity was quantified. First, the boundary of the nucleus was traced and then superimposed onto the IL-33^+^ image using Fiji software (Schindelin et al., 2012). Then, IL-33^+^ signal intensity per pixel was averaged across the nuclear area. This process was used to compare Olig2^+^ cells and S100β^+^ cells in the M2. Additionally, the percentages of S100β^+^, Olig2^+^, and other cells among IL-33-expressing cells were quantified across brain regions. The percentages of c-Fos-expressing cells among NeuN^+^ neurons were also quantified. The correlation between the number of IL-33^+^ cells and the fold change of c-Fos^+^ neurons in WT mice normalized with *Il33* ^−/−^ mice was analyzed in the brain areas where a significant increase in c-Fos expression was observed in *Il33* ^−/−^ mice immediately after EPM. c-Fos fold change in WT mice was calculated as follows: % c-Fos^+^ neurons (NeuN^+^ cells) in WT mice after EPM/the average % c-Fos^+^ neurons in *Il33* ^−/−^ mice after EPM. The correlation between the % c-Fos^+^ neurons and the number of IL-33^+^ cells in WT mice was analyzed immediately after EPM. Additionally, the number of IL-33-expressing cells was analyzed in WT mice with and without the EPM. These analyses were performed blind to mouse genotype.

#### ELISA

Murine IL-33 concentration in serum was determined using DuoSet ELISA kits (R&D Systems, catalog #DY3626-05) following the manufacturer’s standard protocol.

### Quantitative reverse transcription (qRT)-PCR analysis

qRT-PCR was performed following a standard protocol. Total RNA was isolated using RNeasy Plus Micro kit (Qiagen), and cDNA was synthesized from using Superscript III kit (Invitrogen) with oligo(dT)_20_ primers. qPCR was conducted with Maxima SYBR Green/ROX Master Mix (Thermo Fisher Scientific) on ABI 7900HT system (Applied Biosystems). Gene-specific primer sets were obtained from PrimerBank (https://pga.mgh.harvard.edu/primerbank; Spandidos et al., 2010) or designed by using Primer-BLAST (https://www.ncbi.nlm.nih.gov/tools/primer-blast/). The primer sequences are as follows: mouse IL-33, 5’-TCCAACTCCAAGATTTCCCCG-3’ and 5’-CATGCAGTAGACATGGCAGAA-3’; mouse β-actin, 5’-CCTGTATGCCTCTGGTCGTA-3’ and 5’-CCATCTCCTGCTCGAAGTCT-3’.

### Western blotting

Frontal cortex tissues were sectioned and lysed in RIPA buffer containing 0.1% SDS, a protease inhibitor cocktail (Roche), and a phosphatase inhibitor (Sigma Aldrich). Tissue samples were prepared on NuPAGE Bis-Tris Mini Gel (Life Technologies), followed by the transfer to PVDF membrane (Millipore) following a standard protocol. After blocking in 5% skim milk/PBS/0.1% Tween® 20 (PBS-T) for 1 h, the membrane was incubated with the primary antibody overnight at 4°C and then incubated with the secondary andibody for 1 h at room temperature. Gel images were captured by ImageQuant LAS 4000 LAS 4000 mini (GE Healthcare) and analyzed by ImageJ (Schneider et al., 2012). The following primary antibodies were used: goat anti-mouse IL-33 (R&D Systems, RRID: AB_884269), diluted 1:1000, and mouse anti-β-actin, diluted 1:5000 (Santa Cruz Biotechnology, RRID: AB_626632).

### Statistical analysis

Data were analyzed using Student’s *t* test, one-way ANOVA, Kruskal–Wallis test, and two-way repeated measures ANOVA (two-way RM ANOVA) using GraphPad Prism 7 (GraphPad Software) and SigmaStat 4.0 (Systat Software). *Post hoc* analyses for one-way ANOVA, Kruskal–Wallis test, and two-way RM ANOVA were performed using Tukey’s, Dunnett’s, and Sidak’s method, respectively. Significant differences were accepted at *p* < 0.05. All data are presented as mean ± SEM.

## Results

### Localization of IL-33 to the nuclei of oligodendrocyte-lineage cells and astrocytes in the adult mouse brain

Although previous studies have shown that IL-33 is mostly expressed in the nuclei of oligodendrocytes in the adult mouse brain (Gadani et al., 2015), it is not clear whether IL-33 shows a similar expression pattern across brain regions. We assessed the expression patterns of IL-33 with immunohistochemistry in several distinct regions of the adult mouse brain (Fig. 1). First, we compared IL-33 expression patterns between *Il33* ^−/−^ and WT mice to verify the specificity of anti-IL-33 antibody. The results showed that IL-33 immunostaining was mainly colocalized to the cell nucleus and did not show any nonspecific signals (Fig. 1*A*,*B*). IL-33 proteins were expressed constitutively in the brains of WT mice. In contrast, IL-33 proteins were not detected in the peripheral blood of WT mice under physiological conditions (Fig. 1C). We then assessed IL-33 expression and its colocalization to specific cell types in various brain regions (Fig. 1*D*,*E*,*G*,*H*). Consistent with previous studies, IL-33-expressing cells were most abundant in white matter, such as the CC (Fig. 1*D*). IL-33-expressing cells were also distributed throughout the superficial layers of the cortex, e.g., medial prefrontal cortex (mPFC), piriform cortex (PCX), periventricular hypothalamic nucleus (Pe), central amygdala (CeA), basolateral amygdala (BLA), cortical amygdala (CoA), and hippocampus (Fig. 1*D*). At the cellular level, IL-33 was mainly colocalized to the nuclei of both Olig2^+^ oligodendrocyte-lineage cells and S100β^+^ astrocytes (Fig. 1*E*). Higher IL-33 signals were observed in Olig2^+^ cells compared to S100β^+^ astrocytes as shown in the M2 (Fig. 1*F*). Notably, we found that IL-33 colocalization patterns varied across brain regions (Fig. 1*G*). In the CC and M2, IL-33 was predominantly expressed in Olig2^+^ cells (Fig. 1*E*,*G–I*). In the mPFC, IL-33 was expressed in both Olig2^+^ cells and S100β^+^ astrocytes (Fig. 1*E*,*G–I*). In the Pe, BLA, and CoA, IL-33 was predominantly expressed in S100β^+^ astrocytes (Fig. 1*E*,*I–K*). Although IL-33 signals did not generally localize to neurons (NeuN^+^ cells) or microglia (Iba1^+^ cells; Fig. 1*J*), a small group of IL-33^+^ neurons were detected in the granular cell layer of ventral dentate gyrus (vDG; Fig. 1*K*). These results suggest that IL-33 expression either represents subpopulations of Olig2^+^ and S100β^+^ cells or is modified by local neuronal activity. Thus, our detailed analysis of IL-33 expression in subregions of the brain identified its complex expression pattern in the adult mouse brain, underscoring a fundamental role of IL-33 in brain function and behavior.

### Reduced anxiety-like behaviors in *Il33* ^−/−^ mice

**Figure 2.**
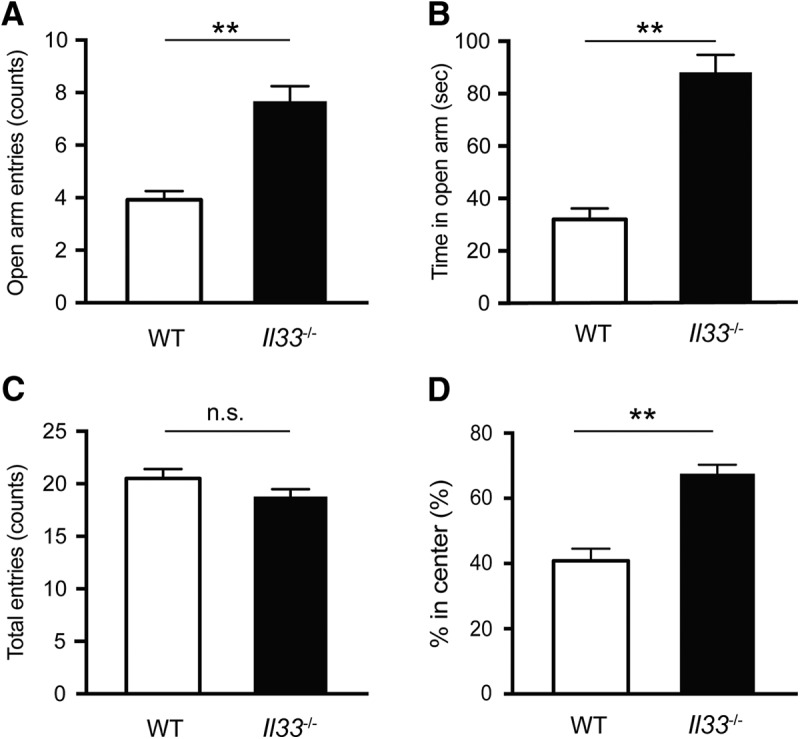
Reduced anxiety-like behavior in *Il33* ^−/−^ mice. ***A***, Increased open arm entries into the EPM. ***B***, Increased time spent in the open arms in the EPM. ***C***, No difference in total entries between WT and *Il33* ^−/−^ mice in the EPM. WT mice, *n* = 12; *Il33* ^−/−^ mice, *n* = 9. ***D***, Increased time spent in the center during the OFT. WT mice, *n* = 9; *Il33* ^−/−^ mice, *n* = 7. Each bar represents mean ± SEM; n.s, not significant; **p < 0.01 (Student’s *t* test; see Table 2 for the detail of statistical analysis).

Previous studies on postmortem brains of patients with mood disorders revealed alterations in the numbers of oligodendrocytes and astrocytes, and their gene expression patterns (Hamidi et al., 2004; Uranova et al., 2004; Aston et al., 2005; Edgar and Sibille, 2012; Nagy et al., 2015). Evidence obtained in rodent studies also supports the involvement of oligodendrocytes and astrocytes in anxiety-related behaviors. Genetic manipulation of several oligodendrocyte-related genes, such as *Cnp1* and *Plp1*, led to altered anxiety-related behaviors (Tanaka et al., 2009; Edgar et al., 2011). Loss of astrocytes in the prefrontal cortex was also shown to induce anxiety-like behaviors in rats (Banasr and Duman, 2008). Given the enriched expression of IL-33 in oligodendrocyte-lineage cells and astrocytes, we assessed the impact on *Il33* deficiency on anxiety-related behaviors. As shown in Figure 2*A*,*B*, *Il33* ^−/−^ mice entered the open arms more frequently and spent more time in the open arms in the EPM. There was no significant difference between WT and *Il33* ^−/−^ mice in total number of entries (Fig. 2*C*). Consistent with these findings, *Il33* ^−/−^ mice spent more time in the center area of the open field compared to WT mice in a novel environment using a 10-min OFT (Fig. 2*D*). These findings show that *Il33* ^−/−^ mice exhibit reduced anxiety-like behaviors, suggesting that the absence of IL-33 may influence neuronal function relevant for anxiety-related behavior.

### Increased neuronal c-Fos immunoreactivity in brain regions linked to anxiety-related behavior in *Il33* ^−/−^ mice

**Figure 3.**
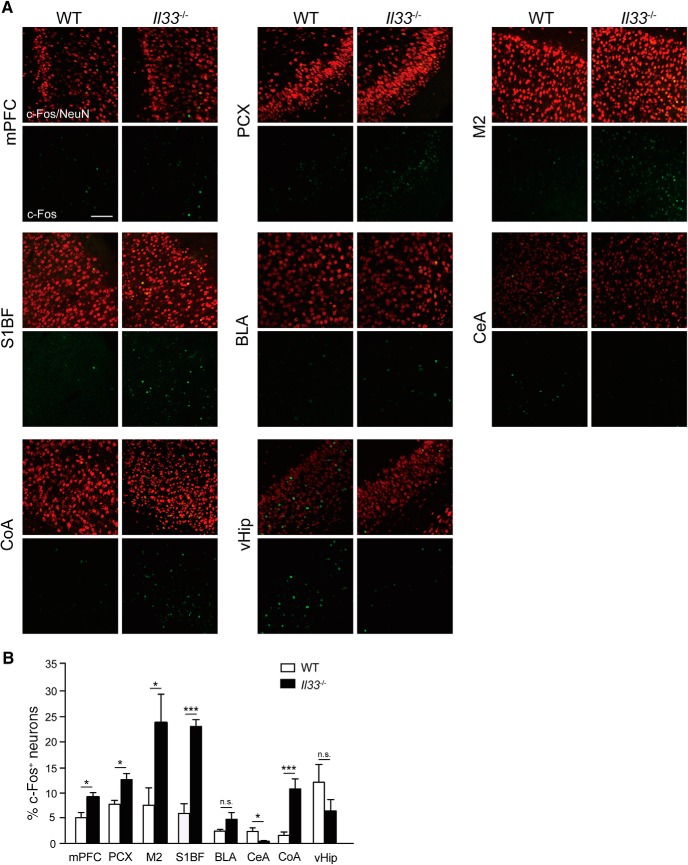
Altered c-Fos immunoreactivity in brain regions related to anxiety in *Il33* ^−/−^ mice. ***A***, Representative images of c-Fos expression in NeuN^+^ neurons. Location of each brain region is indicated in Fig. 1*D* [***a***, mPFC; ***b***, M2; ***d***, PCX; ***e***, S1BF; ***g***, CeA; ***h***, BLA; ***i***, CoA; ***k***, ventral hippocampus (vHip)]. ***B***, Quantification of c-Fos expression in various brain regions. Percentages of c-Fos-expressing neurons among all neurons (NeuN^+^ cells) were calculated and compared between WT and *Il33* ^−/−^ mice (*n* = 3-4). Scale bar, 100 µm. Each bar represents mean ± SEM; n.s., not significant; *p < 0.05, **p < 0.01 (Student’s *t* test; see Table 2 for the detail of statistical analysis).

To understand the impact of *Il33* deficiency on neuronal activity, we determined whether c-Fos immunoreactivity, an indicator of neuronal activity, was altered in brain regions implicated in anxiety-related behaviors (Silveira et al., 1993; Duncan et al., 1996; Linden et al., 2003; Linden et al., 2004). The number of c-Fos-expressing neurons (c-Fos^+^ NeuN^+^ cells) was altered in *Il33* ^−/−^ mice compared to WT mice in various brain regions after the EPM (Fig. 3). The prominent increase in c-Fos expression was observed in the M2, CoA, anterior olfactory area, and somatosensory cortex in *Il33* ^−/−^ mice (Fig. 3*A*,*B*; data not shown). As for the areas associated with anxiety-related behavior, increased neuronal c-Fos expression was observed in the mPFC and CoA while decreased neuronal c-Fos expression was detected in the CeA (Fig. 3*A*,*B*). These findings demonstrated that *Il33* deficiency led to altered neuronal activity in various brain regions, including those related to anxiety.

### Relationship between IL-33 expression and neuronal activity

**Figure 4.**
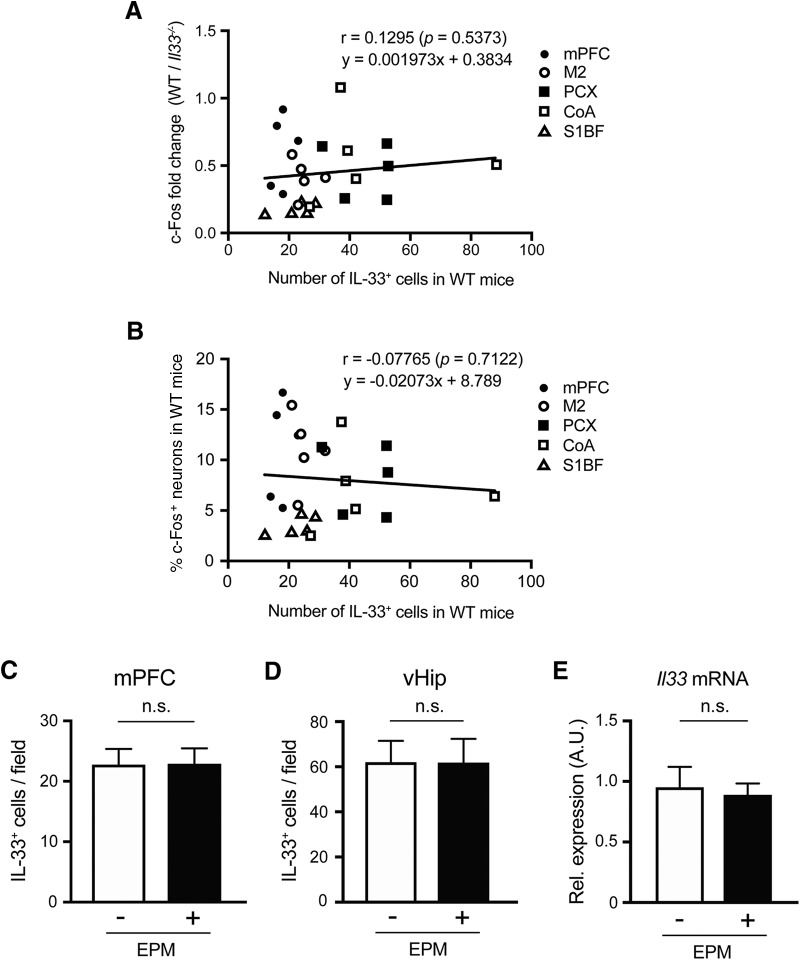
Relationship between IL-33 expression and c-Fos immunoreactivity. ***A***, Correlation between the number of IL-33^+^ cells per field in WT mice and fold change of % c-Fos^+^ neurons (NeuN^+^ cells) per field in WT mice normalized with average % c-Fos^+^ neurons per field in *Il33* ^−/−^ mice. Each dot refers to the brain region of an individual mouse (WT mice, *n* = 5; *Il33*^−/−^ mice, *n* = 5). ***B***, Correlation between the % c-Fos^+^ neurons per field in WT mice and the number of IL-33^+^ cells per field in WT mice based on c-Fos elevated brain regions in Il33^−/−^ mice after EPM. Each dot refers to the brain region of an individual mouse. ***C***, ***D***, No difference in IL-33 expression in the mPFC and ventral hippocampus (vHip) between WT mice with and without the EPM (EPM^+^ and EPM^–^). ***E***, No difference in the *Il33* mRNA expression in the fontal cortex between EPM^+^ and EPM^-^. EPM^+^ *n* = 4, EPM^–^ *n* = 4. A.U., arbitrary unit. Each bar represents mean ± SEM; n.s., not significant (Pearson correlation, Student’s *t* test; see Table 1 for the detail of statistical analysis).

We next determined whether IL-33 expression and neuronal activity influence one another. As described above, c-Fos immunoreactivity was significantly increased in the mPFC, PCX, M2, somatosensory cortex, barrel field (S1BF), and CoA regions of *Il33* ^−/−^ mice after the EPM (Fig. 3*B*). Thus, we analyzed the correlation between expression of IL-33 in these areas in WT mice and fold change of c-Fos^+^ neurons in *Il33* ^−/−^ mice compared to WT mice. We did not observe a correlation between the number of IL-33^+^ cells and the fold change of c-Fos^+^ neurons in WT mice normalized with *Il33* ^−/−^ mice in these regions after the EPM (Fig. 4*A*). We also analyzed the correlation between % c-Fos^+^ of NeuN^+^ neurons and IL-33 expression of WT mice after the EPM, but did not observe any correlation (Fig. 4*B*). We then examined the influence of the EPM experiment, a stressful paradigm that induces neuronal activities, on the levels of IL-33 expression. We did not observe any significant difference in the number of IL-33-expressing cells in several brain areas in mice with or without the EPM (Fig. 4*C*,*D*). qRT-PCR analysis of *Il33* mRNA expression also confirmed these findings (Fig. 4*E*). Thus, our data suggest that *Il33* deficiency widely affects neuronal activity throughout the brain, but that the levels of IL-33 expression and local neuronal activities are not directly related.

### Intact sociability and impaired social recognition in *Il33* ^−/−^ mice

**Figure 5.**
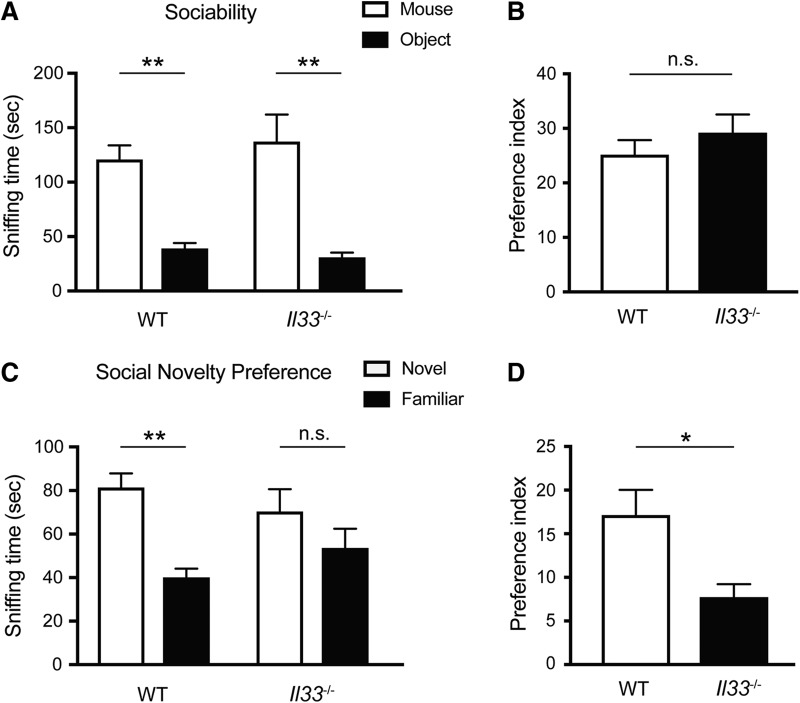
Altered social behaviors in *Il33* ^−/−^ mice. ***A***, No significant difference in sociability between WT and *Il33* ^−/−^ mice. (*F_genotype x chamber_*_(1,22)_ = 0.9178, *p* = 0.3485; *F_genotype_*_(1,22)_ = 0.09669, *p* = 0.7588; *F_Chamber_*_(1,22)_ = 54.44, *p* < 0.001). Both WT and *Il33* ^−/−^ mice preferred mice to objects (**p < 0.01, *post hoc* Sidak test). ***B***, Preference index data for sociability behaviors (*p* = 0.3587, Student’s *t* test). ***C***, Reduced preference to novel mice in *Il33* ^−/−^ mice. WT and *Il33* ^−/−^ mice differed significantly in preference trial (*F_genotype x chamber_*_(1,22)_ = 4.681, *p* < 0.05; *F_genotype_*_(1,22)_ = 0.00224, *p* = 0.8824; *F_Chamber_*_(1,22)_ = 26.34; *p* < 0.0001). Only WT mice showed a significant preference to novel mice (**p < 0.01, *post hoc* Sidak test). ***D***, Preference index data for social novelty preference. *Il33* ^−/−^ group showed a significantly lower preference index than the WT group (*p < 0.05, Student’s *t* test). Each bar represents mean ± SEM; n.s., not significant. See Table 2 for the detail of statistical analysis.

To address whether IL-33 deficiency specifically influences anxiety-related behaviors, we also assessed the social behaviors of *Il33* ^−/−^ mice in the three-chamber social interaction test (Moy et al., 2004; Gkogkas et al., 2013; Lipina et al., 2013; Filiano et al., 2016). In the sociability trial, where a test mouse spends time interacting with either an object or another mouse, *Il33* ^−/−^ mice showed a clear preference to the mouse, the same behavior as WT mice (Fig. 5*A*,*B*). In contrast, in the social novelty preference trial, where a test mouse interacts with either a novel mouse or a familiar mouse, *Il33* ^−/−^ mice did not show an increase in interaction with the novel mouse, as was observed in WT mice (Fig. 5*C*,*D*). These results suggest that IL-33 is also required for social novelty recognition. Social novelty recognition is known to be affected by olfactory impairment (Spehr et al., 2006), and IL-33 is expressed in the PCX, one of the main components of the olfactory system. Thus, IL-33 deficiency may impair social novelty recognition, via dysregulation of neuronal circuits and/or olfaction.

## Discussion

In this study, we reported a novel role of the nuclear alarmin IL-33 in the regulation of anxiety-related and social behaviors in mice. We found that *Il33* deficiency resulted in anxiolytic behaviors in two well-established behavioral paradigms, the OFT and the EPM. Consistent with these behavioral changes, we observed that the expression of an immediate early gene, *c-Fos*, was significantly altered in brain regions related to anxiety in *Il33* ^−/−^ mice. *Il33* ^−/−^ mice also showed impaired social recognition in the three-chamber social interaction test. In addition, we found that IL-33 expression patterns considerably varied across different brain regions in adult mice and that subsets of Olig2^+^ oligodendrocyte-lineage cells and S100β^+^ astrocytes did not express IL-33. Thus, IL-33 expression may represent glial subpopulations whose distribution varies between brain regions. Although IL-33 expression was detected in the adult brain, there was no correlation between IL-33 expression and c-Fos immunoreactivity changes. Thus, our study suggested that *Il33* deficiency may dysregulate the development and/or maturation of multiple neuronal circuits.

What neuronal circuitry is affected by *Il33* deficiency? The neural circuitry underlying anxiety-related behaviors is believed to consist of stratified structures, from detection (e.g., thalamus, sensory cortex), to interpretation (e.g., amygdala, hippocampus, LS), evaluation (e.g., mPFC, NAc, hypothalamus, VTA), and response (e.g., motor cortex, brainstem; Silveira et al., 1993; Linden et al., 2003; Salomé et al., 2004; Sul et al., 2011; Tye et al., 2011; Huang et al., 2013; Likhtik et al., 2014; Calhoon and Tye, 2015). On the other hand, the brain areas underlying social recognition or social memory are reported as olfactory bulb, medial amygdala, entorhinal cortex, perirhinal cortex and hippocampus (Camats Perna and Engelmann, 2017). Thus, it is unlikely that the behavioral alterations of *Il33* ^−/−^ mice were explained by some common neuronal circuits where IL-33 signaling plays a critical modulatory role. Rather, it is speculated that *Il33* deficiency dysregulates the development and/or maturation of multiple neuronal circuits relevant to anxiety and social behaviors. Our data that IL-33 expression is neither modified by behaviors nor correlated with c-Fos staining patterns in the adult brain support this hypothesis. Previous studies showed that IL-33 expression dynamically changed across various brain regions during the postnatal period (Wicher et al., 2013). Future studies are required to address the impact of *Il33* deficiency in different brain areas at different developmental stages on anxiety-related and social behaviors.

The mechanisms by which IL-33 influences the development and/or maturation of multiple neuronal circuits linked to anxiety and social recognition-related behaviors are not clear at this moment. Previous studies reported that alterations in oligodendrocytes and astrocytes impair these behaviors in mice. Studies on mice with mutations in oligodendrocyte-related genes, such as *Cnp1* and *Plp1*, and a mouse model of cuprizone-induced demyelination suggest that the loss of integrity of small-diameter myelinated axons due to oligodendrocyte dysfunction may be a common mechanism underlying reduced anxiety-like behaviors (Ganter et al., 1999; Evangelou et al., 2001; Lappe-Siefke et al., 2003; Edgar et al., 2004; 2009; 2011; Tanaka et al., 2009; Xu et al., 2009). In addition, mice deficient in CD38, which regulates astrocyte and oligodendrocyte maturation (Hattori et al., 2017), are reported to show impaired social recognition (Kim et al., 2016). Loss of astrocytes in the prefrontal cortex has been shown to induce anxiety-like behaviors in rats (Banasr and Duman, 2008). Thus, there are at least three potential mechanisms by which IL-33 affects anxiety and social novelty recognition. First, IL-33 in oligodendrocytes may be required for the maintenance of small-diameter axons and thus neuronal connections across different brain regions related to these behaviors. Second, IL-33 may be required for the development and maturation of astrocytes and/or the proper astrocytic modulation of neuronal activities. Third, IL-33 may play the abovementioned roles both in oligodendrocytes and astrocytes, which together contribute to anxiety and social novelty recognition. Finally, it needs to be determined whether IL-33 functions as a cytokine or a transcriptional modulator. If IL-33 functions as a cytokine, the effect is mediated via its receptor ST2. Because ST2 is expressed in microglia in the adult brain (Yasuoka et al., 2011; Yang et al., 2017), altered microglia-neuron interactions may also underlie neuronal dysfunction and behavioral abnormalities. If IL-33 functions as a transcriptional modulator, altered glial cell functions in *Il33* ^−/−^ mice can be explained by changes in gene expression patterns. Further studies are required to address these various possibilities to understand the mechanisms by which IL-33 influences brain development and/or maturation.

It is likely that various behaviors may be profoundly impaired in *Il33* ^−/−^ mice, because IL-33 has been reportedly expressed in various brain regions and dynamically changed throughout brain development (Wicher et al., 2013). In particular, social novelty recognition is known to be affected by olfactory impairment (Spehr et al., 2006). Given that c-Fos immunoreactivity was altered in the brain regions related to sensory processing in *Il33* ^−/−^ mice, altered social novelty recognition in *Il33* ^−/−^ mice may have been caused by general impairment of olfactory sensory processing. Alternatively, social novelty recognition deficits may indicate general impairment of recognition memory in *Il33* ^−/−^ mice. Further behavioral assessments, including olfaction and memory tests are required to address these issues in future studies. As discussed above, systematic experiments using cell-type-specific deletion of the *Il33* gene in WT mice and/or recovery of IL-33 expression in *Il33* ^−/−^ mice during brain development and in adulthood are necessary to clarify the exact role of IL-33 in these behaviors.

IL-33 is also expressed outside of the brain. Although the level of IL-33 in the sera of WT mice was below the ELISA assay detection threshold, it is possible that a subthreshold level of IL-33 circulates in the blood and reaches the brain to influence its function and behavior. In addition, *Il33* deficiency in peripheral organs may affect the brain via another mediator (e.g., another cytokine, metabolites, neural connections). One recent study revealed that IL-33 regulates gut microbiota homeostasis by promoting IgA production from B cells (Malik et al., 2016). Because germ-free mice, which lack gut microbiota, have been shown to exhibit less anxiety-like behavior (Diaz Heijtz et al., 2011; Neufeld et al., 2011; Clarke et al., 2013), IL-33 deficiency may cause anxiolytic effects and impairments of social behaviors by altering the composition of gut microbiota. IL-33 has also been shown to maintain the immune function of meninges (Gadani et al., 2016), which can influence brain development by secreting trophic factors (Siegenthaler et al., 2009; Lehtinen and Walsh, 2011). Future studies on brain-specific deletion of the *Il33* gene will address the contribution of central and peripheral IL-33 to anxiety-related and social behaviors.

An increasing number of studies report that day/night cycle significantly affects behavioral phenotypes of rodents that are nocturnal (Roedel et al., 2006; Fonken et al., 2009; Sidor et al., 2015). Particularly, emotional and stress-related behaviors are known to be affected by day/night cycle (Fonken et al., 2009; Leach et al., 2013; Tapia-Osorio et al., 2013; Griesauer et al., 2014; Ben-Hamo et al., 2016). While the findings under an inactive phase of rodents in a nonreversed day/night cycle provide new biological insights into their behaviors, the analysis of their behaviors under the active phase in a reversed day/night cycle will be helpful to determine the biological significance of the findings. Future studies of *Il33* ^−/−^ mice under an active phase will be important to confirm the current findings and identify additional behavioral abnormalities.

## Synthesis

Reviewing Editor: Cheryl Sisk, Michigan State University Decisions are customarily a result of the Reviewing Editor and the peer reviewers coming together and discussing their recommendations until a consensus is reached. When revisions are invited, a fact-based synthesis statement explaining their decision and outlining what is needed to prepare a revision will be listed below. The following reviewer(s) agreed to reveal their identity: Martha Havenith, Lindsay De Biase.

Reviewers noted that the revised manuscript is substantially improved and that most issues raised in the previous review have been adequately addressed. However, there are still some places in which conclusions drawn about the role of IL-33 on behavior go beyond the experiments that were actually conducted, and the reviewers offered additional suggestions for revisions. Please address these concerns in a revised manuscript, and particularly tone down the statements referred to in Item 1.

1. Published data show that IL-33 levels are higher during postnatal development (Wicher et al. 2013), suggesting a prominent role in development. The lack of correlation between brain regions that express high levels of IL-33 and those that show the largest changes in c-FOS (Fig. 4) in IL-33 KO mice suggests the absence of a causal relationship between adult levels of IL-33 and patterns of neuronal activity, and, rather, that perturbed circuit maturation accounts for altered patterns of neuronal activity. The fact that social novelty detection is also impaired in IL-33 KO mice raises the possibility that there are general learning and memory deficits in these mice and indicates that behavioral alterations are not specific to anxiety-related behaviors. This, too, argues that there are broad changes in circuit function due to lack of IL-33 during development rather than an acute role for IL-33 in regulating these behaviors in the adult. Finally, the lack of change in # of IL-33+ cells or IL-33 expression levels in mice undergoing EPM, suggests that Il-33 levels are not dynamically regulated in the adult in the absence of a more profound pathological stimulus.

Together this means that the authors still cannot claim “our study suggests that IL-33 influences anxiety-related and social behaviors by modulating neuronal activities in relevant brain regions” (abstract). And they cannot claim that “our findings have shed a new light on the role of IL-33 on multiple behaviors under physiological conditions” (discussion). To make these claims, the study must have some attempt to manipulate IL-33 levels within the adult to assess the impact on neuronal activity and anxiety like behaviors. In the absence of such data, the study strongly suggests that “IL33 deficiency dysregulates the development and/or maturation of multiple neuronal circuits relevant to anxiety and social behaviors” (discussion lines 337-339).

2. Title, abstract and significance statement: Make the two behavioral assays (anxiety and sociability) equivalent. Right now you are first emphasizing anxiety, and then adding sociability on at the end of the section. I would change that to e.g. 'Notably, Il33-/- mice exhibited reduced anxiety-like behaviors in the elevated plus maze and the open-field test, as well as deficits in social novelty recognition, despite their intact sociability, in the three-chamber social interaction test.'

3. It is quite likely that other behaviors apart from anxiety and sociability are affected (see points of improvement for the Discussion section). Even though these could not (and don't need to) be tested in the current study, it is important to point out throughout the text that behavioral changes are not confined to the two tested domains. For instance, I would suggest to change the title to something more neutral like 'Behavioral changes in mice lacking IL33' or something along those lines.

4. Methods: For the EPM and sociability test, add information on how many observers rated each video. Also, to give an estimate of inter-rater reliability you only need to do pair-wise cross-correlations between the scores given by different observers, and give the average correlation in the text. So I would add that - it settles a niggling problem, and depending on how you store your data, this should take between 10 minutes and a couple of hours to compute.

5. Results: Fig. 4a - Wouldn't it be more useful to do this per IL33-/- mouse (always normalized by average fold change in all WT mice)? The low number of data points in the graph makes it unlikely that one would see a pattern even if there was one. Brain areas can be shown as dots of different colors or the like (as is actually being done in 4b). Turn x and y axis in 4B around so that 4A and 4B match each other.

6. Discussion: Add a paragraph discussing that given the wide distribution of IL33 expression there may be further behavioral consequences which you couldn't test here. In particular, what do we know about olfactory impairments in IL33-/- mice -given that IL33 cells are expressed in piriform cortex? That could in fact explain the impaired social recognition - their social abilities are perfectly fine, but their impaired sense of smell prevents them from distinguishing familiar and new mice in the context of the three-chamber task (where other forms of interaction are limited). Consistently with this, reformulate Line 312 to allow for the possibility that social recognition behavior may be impaired via different routes that have more or less to do with social interaction versus sensory processing. On a related note, do we know anything about locomotion/somatosensory abilities in these mice?
